# Transcriptomic profiling of TK2 deficient human skeletal muscle suggests a role for the p53 signalling pathway and identifies growth and differentiation factor-15 as a potential novel biomarker for mitochondrial myopathies

**DOI:** 10.1186/1471-2164-15-91

**Published:** 2014-02-01

**Authors:** Susana Graciela Kalko, Sonia Paco, Cristina Jou, Maria Angels Rodríguez, Marija Meznaric, Mihael Rogac, Maja Jekovec-Vrhovsek, Monica Sciacco, Maurizio Moggio, Gigliola Fagiolari, Boel De Paepe, Linda De Meirleir, Isidre Ferrer, Manel Roig-Quilis, Francina Munell, Julio Montoya, Ester López-Gallardo, Eduardo Ruiz-Pesini, Rafael Artuch, Raquel Montero, Ferran Torner, Andres Nascimento, Carlos Ortez, Jaume Colomer, Cecilia Jimenez-Mallebrera

**Affiliations:** 1 Bioinformatics Core Facility, IDIBAPS, Hospital Clinic, Barcelona, Spain; 2 Neuromuscular Unit, Neurology Department, Fundación Sant Joan de Déu, Hospital Sant Joan de Déu, Barcelona, Spain; 3 Pathology Department, Hospital Sant Joan de Déu, Barcelona, Spain; 4 Institute of Anatomy, Faculty of Medicine, University of Ljubljana, Ljubljana, Slovenia; 5 Department of Child, Adolescent, and Developmental Neurology, Children’s Hospital, University Medical Centre Ljubljana, Ljubljana, Slovenia; 6 U.O.S. Diagnostica Malattie Neuromuscolari, Fondazione Ospedale Maggiore Mangiagalli e Regina Elena, IRCCS, Milan, Italy; 7 Laboratory for Neuropathology, Ghent University Hospital, Ghent, Belgium; 8 Institute of Neuropathology, Hospital de Bellvitge, Barcelona, Spain; 9 Neuropaediatrics Department, Vall d’Hebron Hospital, Barcelona, Spain; 10 Biochemistry and Molecular Biology Department, University of Zaragoza, Zaragoza, Spain; 11 Center for Biomedical Research on Rare Diseases (CIBERER), Instituto de Salud Carlos III, Madrid, Spain; 12 Clinical Biochemistry Department, Hospital Sant Joan de Déu, Barcelona, Spain; 13 Orthopaedic Surgery & Traumatology Department, Hospital Sant Joan de Déu, Barcelona, Spain

**Keywords:** Gene expression, Microarrays, Bioinformatics, Mitochondrial DNA, Mitochondrial DNA depletion, Mitochondrial encephalomyopathy, Thymidine kinase 2, Skeletal muscle, p53, Apoptosis, GDF-15

## Abstract

**Background:**

Mutations in the gene encoding thymidine kinase 2 (TK2) result in the myopathic form of mitochondrial DNA depletion syndrome which is a mitochondrial encephalomyopathy presenting in children. In order to unveil some of the mechanisms involved in this pathology and to identify potential biomarkers and therapeutic targets we have investigated the gene expression profile of human skeletal muscle deficient for TK2 using cDNA microarrays.

**Results:**

We have analysed the whole transcriptome of skeletal muscle from patients with *TK2* mutations and compared it to normal muscle and to muscle from patients with other mitochondrial myopathies. We have identified a set of over 700 genes which are differentially expressed in TK2 deficient muscle. Bioinformatics analysis reveals important changes in muscle metabolism, in particular, in glucose and glycogen utilisation, and activation of the starvation response which affects aminoacid and lipid metabolism. We have identified those transcriptional regulators which are likely to be responsible for the observed changes in gene expression.

**Conclusion:**

Our data point towards the tumor suppressor p53 as the regulator at the centre of a network of genes which are responsible for a coordinated response to TK2 mutations which involves inflammation, activation of muscle cell death by apoptosis and induction of growth and differentiation factor 15 (GDF-15) in muscle and serum. We propose that GDF-15 may represent a potential novel biomarker for mitochondrial dysfunction although further studies are required.

## Background

Mitochondrial myopathy and mitochondrial encephalomyopathy are terms that define a group of neurological disorders characterized by defective oxidative phosphorylation [1-3]. Multiple organs may be affected including skeletal muscle, liver and central nervous system. The genetics and pathogenesis of mitochondrial disorders are complex because of the interaction of the mtDNA and nuclear genomes. The mtDNA contains 37 genes encoding 13 mitochondrial proteins. The remaining of the over 1500 predicted mitochondrial proteins are encoded by the nuclear genome [4]. The majority of these nuclear genes are unknown (over 50 are known as disease causative genesmaking it very difficult to diagnose at the molecular level mitochondrial disorders not due to mtDNA mutations [5,6].

Mitochondrial DNA depletion syndrome (MDS) is a clinically and genetically heterogeneous subgroup of mitochondrial diseases of nuclear origin which is characterised by severe reduction of mtDNA content in specific tissues, mainly muscle, liver and brain To date 9 nuclear genes have been identified as causing MDS which encode proteins directly involved in the maintenance of the mitochondrial deoxyribonucleoside triphosphate pools (dNTPs) such as Thymidine kinase 2 (TK2), deoxyguanosine kinase (DGOUK), p53 dependent ribonucleotide reductase subunit 2 (RRM2B) and thymidine phosphorylase (TYMP), or in mtDNA replication such as mtDNA polymerase γ (POLG) and Twinkle (C10orf2). The role of the other three MDS causative gene products (MPV17), and the α and β subunits of succinate-CoA ligase (SUCLG1 and SUCLA2 respectively) in mtDNA maintenance is less clear [7].

Clinically, MDS are divided into myopathic, hepatocerebellar or encephalomyopathic, depending on which tissues are mainly affected at the onset of the symptoms. The myopathic form is most frequently associated with mutations in the *TK2*, *SUCLA2* and *RRM2B* genes. Thymidine kinase 2 is responsible for the phosphorylation of pyrimidine nucleosides (deoxythymidine, deoxycytosine and deoxyuridine) inside the mitochondria as part of the salvage pathway. In contrast, thymidine kinase 1 (TK1) participates in the de novo deoxythymidine monophosphate (dTMP) synthesis pathway in the cytoplasm which is then imported into mitochondria. In most reported cases mutations in TK2 result in almost complete absence of enzyme activity and as a consequence in a very severe reduction in mtDNA content (>90%) which leads to combined respiratory chain complexes deficiency. Myopathic MDS due to TK2 defects is characterised by neonatal or early onset of hypotonia which progresses rapidly into a severe myopathy and infantile death because of respiratory failure although there are reports of patients which survive longer [8,9]. Electromyography shows myopathic signs; creatine kinase levels (CK) are markedly elevated and lactate is moderately high. In addition to muscle symptoms patients may also have CNS involvement [7,10-12]. At the pathological level, muscle biopsies frequently show dystrophic changes with loss of muscle fibres, proliferation of connective and adipose tissues, ragged red fibers with mitochondria proliferation and profound cytochrome c oxidase deficiency and increased intracellular lipid content [10]. To date, more than 20 missense mutations, deletions and insertions have been reported in the TK2 gene. Some of them have been reported in more than one non-related families [11,12].

Two mouse models of TK2 defects have been generated. *Tk2* knocked-out mice [13,14] suffer growth retardation and hypothermia and die within 2–4 weeks of life. They show progressive depletion of mtDNA in skeletal muscle, heart and liver. The morphology of skeletal muscle seems unaffected whereas cardiac muscle is disorganised and contains abnormally structured mitochondria. The authors conclude that Tk2 is necessary for deoxythymidine triphosphate (dTTP) synthesis in non-replicating cells, whereas fast-replicating cells rely more heavily on the de novo synthesis pathway. Thus, as tissues mature, mtDNA replication is increasingly affected and mtDNA depletion worsens. Detailed analysis of the neurological features of these mice revealed an ataxic phenotype with progressive loss of mtDNA content, respiratory chain activity and protein content of mitochondrially encoded respiratory chain subunits in brain tissue, which is accompanied by selective loss of neuronal types [13]. The *Tk2* knock-in mice bear the human H126N missense mutation and also showed growth retardation and had a generalized weakness, tremor and impaired gait [15]. Tk2 activity was severely reduced in all tissues tested (3% and 1.7% of normal in skeletal muscle and brain respectively) confirming that the mutation severely disrupts Tk2 function. Brain and spinal cord showed the most prominent mtDNA depletion followed by skeletal muscle, heart and kidney whereas in contrast to the KO model, liver mtDNA content was normal. Activities of the respiratory chain enzymes were selectively reduced in brain and spinal cord whereas they were normal in skeletal muscle. Histological analysis of skeletal muscle was unremarkable. In contrast, the equivalent homozygous mutation in humans resulted in severe myopathy [16]. Thus, neither of the two mouse models replicates the severe muscle phenotype and pathology seen in humans.

How the muscle cell responds to mtDNA depletion, impaired oxidative phosphorylation and severe loss of energy production and how these mechanisms differ between MDS and other mitochondrial myopathies is not well understood. A patient was described which survived into adulthood. Analysis of his two consecutive muscle biopsies revealed an increase in mtDNA content with time which paradoxically was accompanied by a decrease in complex IV activity and severe muscle mass and fibre loss [8,17]. These findings suggest that there may have been a selective loss of the most affected fibres and that a compensatory mechanism may be acting in these patients to maintain mtDNA levels in surviving muscle fibres. Identification of such compensatory pathways would be of great importance to devise potential therapies.

In order to address some of these questions we undertook global gene expression analysis of muscle biopsies from MDS patients with mutations in *TK2* and compared it with that of patients with mtDNA large deletions and aged matched normal muscle. We found a large number of genes whose expression was significantly changed which represent the molecular signature of TK2 deficient human muscle. Our data show that the activation of p53 signalling pathway is relevant to the pathogenesis of TK2 defects and show that energy production via glucose and glycogen metabolism are significantly impaired at the transcriptional level. In contrast, genes known to be part of the starvation response mechanism were increased. We describe the over-expression of growth and differentiation factor 15 (GDF-15) in muscle and serum and its potential use as a novel biomarker of mitochondrial disease.

## Results

### Patients and microarray

In the cDNA microarray we included 4 unrelated patients with MDS and mutations in *TK2* (patients 1, 2, 3 and 4, MDS group), 4 patients with large mtDNA deletions as disease control group (patients 5, 6, 7 and 8, MDEL group) and 4 aged-matched healthy individuals (normal control group) (Table 1). Whole genome was analysed using Agilent Human 8 × 60K arrays. Standard array quality controls were performed showing satisfactory results. Unsupervised bi-clustering analysis considering the Agilent oligonucleotides with the largest variations across the whole experiment showed an appropriate segregation of the different samples. Data has been deposited at the National Centre for Biotechnology Information Gene Expression Omnibus (GEO) database as GEO Series accession number GSE43698.

**Table 1:** Clinical summary of patients (* indicates those patients included in the microarray analysis)

**Patient**	**Onset**	**Main clinical symptoms**	**Age at biopsy**	**Genetics**	**Respiratory chain enzymes activities**	**Muscle pathology**	**References**
*P1	2 y	Hypotonia, weakness, progressive gait impairment. Unable to stand. CK 2200U/L	2 y	96% mtDNA depletion Hom A181V *TK2*	N/A	Dystrophic, severe COX reduction. No RRF. Type 1 predominance	Family 1 P2 Galbiati et al., 2006 [12]
*P2	3 y	Hypotonia, weakness, walking difficulties, exercise intolerance, ptosis, ophthalmoplegia, cerebellar vermis atrophy. CK 523U/L	4 y	92% mtDNA depletion het C108W + L257P *TK2*	Complex I, III and IV deficiency	Dystrophic, COX negative fibres (74%). RRF (68%). Moderate increase intracellular lipid. Type 1 predominance	Family 2 P2 Galbiati et al., 2006 [12]
*P3	2 y	Unstable gait, muscle hypotrophy. CK 800 U/L	2 y	90% mtDNA depletion, Hom A181V *TK2*	Complex I, III, IV and V deficiency	Dystrophic, COX negative fibres. Increased intracellular lipids	Pat E Spinazzola et al., 2009 [11]
*P4	2 y	Unstable gait, muscle hypotrophy. CK 1000U/L	2 y	90% mtDNA depletion, Hom A181V *TK2*	Complex I, III, IV and V deficiency	Dystrophic, COX negative fibres. Increased intracellular lipids	Pat C Spinazzola et al. 2009 [11]
*P5	5 y	Low stature, lactic acidosis hypothyroidism, retinitis pigmentosa.	6 y	6331-13994 (7.7 kb) 64% mtDNA	Normal	RRF	
*P6	7 y	Retinitis pigmentosa leukoenchephalopathy deafness	9 y	11033-15157 70%	CI-CIII/CIII	RRF	Pineda et al., 2006
*P7	30 y	Retinitis pigmentosa deafness, ataxia, myopathy, heart conduction defects.	30 y	6331-13994 77% mtDNA molecules	Normal	RRF	
*P8	15 y	PEO, pigmentary degeneration of retina, complete AV block and myopathy	31 y	8.4 kDa deletion; 35% mtDNA molecules	N/A	COX negative fibres and RRF.	
P9	30 y	Eyelid ptosis	38 y	4.8 kDa mtDNA deletion; 40% molecules	N/A	COX negative fibres and RRF.	
P10	4 m	Delayed motor milestones, hypotonia, dystonia, severe axial muscle weakness, brisk deep tendon reflex and mild spasticity in lower limbs. Neurosensorial deafness. Mild methyl malonic aciduria	7 m	87% mtDNA depletion het. p.G350S + p.G350V *SUCLA 2*	Complex II, III and IV deficiency	Generalized COX reduction. No RRF. Moderate increase intracellular lipids	
P11	1.5 y	Proximal muscle weakness, mildly elevated CK levels.	4 and 5 y	95% mtDNA depletion het. p.T77M + p. R161K *TK2*	Normal	COX negative fibres. Type 1 predominance	Wang et al., 2005; Vilá et al., 2008 [8] and 2010
P12	10 yrs	Exercise intolerance, myalgia, lactic acidosis, eyelid ptosis, CK 400U/L, hypertrophic cardiomyopathy, epilepsy	13 y	A3243G tRNA-LEU mtDNA	N/A	RRF	

Yrs: years; mtDNA: mitochondrial DNA; COX: cytochrome oxidase; RRF: Ragged-red-fibres; CK: serum creatine kinase; PEO: progressive external ophthalmoplegia; CK: creatine kinase; y: years; m: months.

### Genes differentially expressed in mtDNA depleted muscle versus control muscle

#### ***Summary***

Using the statistical test RankProd we identified 336 unique genes that were significantly under-expressed and 437 that were significantly over-expressed at FDR < 0.05 in MDS muscle compared to control muscle (Table 2). The list of differentially expressed unique genes is shown in Additional file 1. The 10 top under-expressed and over-expressed genes in this comparison are listed in Table 3. The fold changes are remarkable, particularly for the induced genes (up to 188 fold increase) whereas they are more moderate for the repressed genes (maximum 10 fold decrease).

**Table 2:** Summary of differential genes in MDS vs control muscle

**MDS vs Control**	**DOWN**	**UP**
FDR < 0.05	336	437
FDR < 0.05 & IPA SKM	268	279

Number of unique genes. MDS: mitochondrial DNA depletion syndrome group. FDR: False Discovery Rate. IPA SKM: genes filtered by expression in skeletal muscle according to Ingenuity Pathway Analysis database.

**Table 3:** Top Ten under-expressed and over-expressed genes in MDS vs control muscle

**Gene name**	**Description**	**FC**	**FDR**
**Under-expressed**			
ATP2A1	ATPase, Ca++ transporting, cardiac muscle, fast twitch 1	-9.1	0
FBP2	Fructose-1,6-bisphosphatase 2	-9.1	0
MYLK2	Myosin light chain kinase 2	-7.1	0
ANKRD23	Ankyrin- repeat domain containing protein 23	-7.1	0
ACTN3	Actinin, alpha 3	-6.9	0
AQP4	Aquaporin 4	-6.2	5.88E-04
GLUL	Glutamine synthase	-5.9	0.002
SMTNL2	Smoothelin-like 2	-6.2	2.86E-04
ARRDC2	Arrestin domain containing 2	-5.8	2.86E-04
TRIM63	Tripartite motif containing 63, E3 ubiquitin protein ligase	-5.5	0.002
PYGM	Glycogen phosphorylase	-4.8	0.001
**Over-expressed**			
GDF15	Growth Differentiation Factor 15	188	0
MYH8	Myosin Heavy Chain Embryonic	119	0
TRIB3	Tribbles homolog 3	47	0
MYH3	Myosin Heavy Chain Neonatal	24	0
SNAR-A3	Small nuclear ILF3/NF90-associated RNA A3	23	0
TNNT2	Troponin T2, cardiac	21	0
DEFB1	Defensin beta-1 malignant brain tumours 1	16	0
TMEM63C	Transmembrane protein 63C	16	0
DMBT1	Deleted in malignant brain tumors 1	11.3	0
COL19A1	Collagen type XIX	11	0

MDS: mitochondrial DNA depletion syndrome group. FC: fold-change; FDR: false discovery rate.

Amongst the top under-expressed genes we found some genes encoding skeletal muscle components characteristic of type II muscle fibres (fast) such as sarcoplasmic reticulum Ca^2+^ ATPase 1 (*ATP2A1*), myosin-light chain kinase 2 (*MYLK2*), actinin-type 3 (*ACTN3*) and aquaporin-4 (*AQP4*). These changes indicate a selective loss of type II fibres. In fact, type 1 predominance has been reported in some of the patients included in our microarray [11,12].

Fructose-1,6-bisphosphatase 2 (*FBP2*) is the gene encoding the muscle specific enzyme responsible for the hydrolysis of fructose-1, 6-bisphosphatase into fructose 6-phosphate and inorganic phosphate during gluconeogenesis. As we will see later we found several other genes involved in glucose and glycogen metabolism amongst the significantly under-expressed genes including glycogen phosphorylase (*PYGM*).

Ankyrin repeat domain-containing protein 23 is a member of the muscle ankyrin repeat protein family (MARP). The protein is localized to the nucleus, functioning as a transcriptional regulator. Expression of this protein is induced during recovery following starvation [18].

Glutamate-ammonia ligase (or gluthamine synthethase, *GLUL*) is responsible for the synthesis of glutamine. GLUL is expressed throughout the body and plays an important role in controlling body pH and removing ammonia from the circulation [19].

The ARRDC2 protein belongs to a family of proteins some of which are involved in ubiquitination by the E3 NEDD4 ligase [20,21]. *TRIM63* encodes for the muscle specific E3 ubiquitin ligase MURF1 which is involved in regulating skeletal muscle fibre atrophy. We and others have found decreased *TRIM63* RNA expression in chronic atrophy and in diseased muscle [21].

Regarding the top over-expressed genes, growth and differentiation factor 15 (*GDF-15*) encodes a cytokine which has been involved in a variety of processes including reduced risk of miscarriage and myocardial infarction [22]. Tribbles-homolog 3 (*TRIB3*) is a pseudokinase which negatively regulates NF-κB signalling and sensitizes cells to TNF and TRAIL induced apoptosis. In addition, expression of *TRIB3* increases during periods of reduced nutrient supply to induce lipolysis [23].

*MYH3*, *MYH8* and *TNNT2* encode for the neonatal, embryonic myosin heavy chain and cardiac troponin 2 respectively and are markers of muscle fibre regeneration.

*COL19A1* encodes for the alpha-1 chain of collagen XIX which we have found similarly over-expressed in various dystrophies including Duchenne muscular dystrophy (unpublished observation) and probably reflects the amount of fibrosis observed in the muscle biopsies of these patients [11,12].

#### ***Skeletal muscle expression***

To ascertain which differentially expressed genes are known to be expressed in skeletal muscle we filtered the gene dataset (FDR < 0.05) using Ingenuity Pathways (IPA)-knowledge base (which is based on GNF body atlas and literature findings). Among the 336 under-expressed genes, 268 passed this filter and 279 among the 437 over-expressed genes (Table 2). Using this filter we lost 2 genes in the top 10 over-expressed genes (*SNAR-A3, TMEM63C*). All the top 10 under-expressed genes in the whole set of genes passed the IPA filter. Thus, there was very good agreement between the results of the analysis for the whole set of genes and for the skeletal muscle filtered one. However, IPA may discard some genes with low basal expression levels in skeletal muscle, genes that are tightly developmentally regulated or have not been described as expressed in skeletal muscle. For this reason we decided to focus on the whole set of genes in order to avoid loosing valuable data.

#### ***Gene ontology***

**Figure 1:**
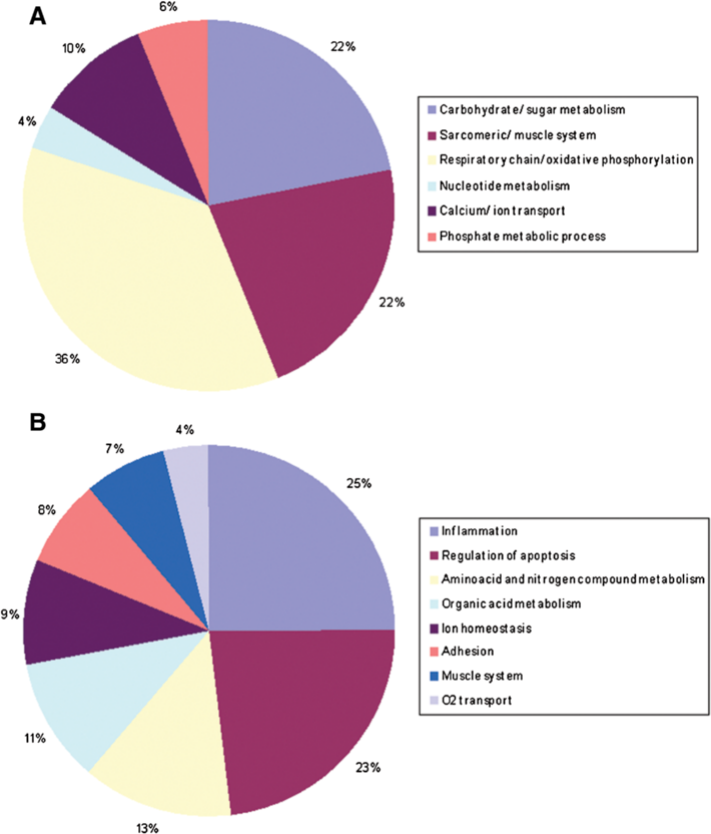
Pie charts representing GO BP categories for (A) under-expressed and (B) over-expressed genes in MDS compared to control muscle.

In order to obtain Gene Ontology enrichment analysis, the lists of differentially over and underexpressed genes (FDR < 0.05) were considered in the tool DAVID [24]. Those GO_BP terms (Gene Ontology Biological Process) with a%FDR < 20 were considered as significantly enriched and selected for further analysis (49GO_BPs for the over-expressed genes and 91 for the under-expressed genes, Additional file 2). For simplicity, we grouped the various GO terms in broader GO_BP categories as shown in Figure 1 (Additional file 2). The GO_BP classes corresponding to the under-expressed genes (Figure 1A) were mainly related to carbohydrate metabolism, respiratory chain and oxidative phosphorylation, nucleotide metabolism and muscle contraction whereas over-expressed genes were mainly associated with inflammation, apoptosis, aminoacid and nitrogen compound metabolism, organic acid synthesis, adhesion, ion homeostasis, muscle system and oxygen transport (Figure 1B).

**Figure 2:**
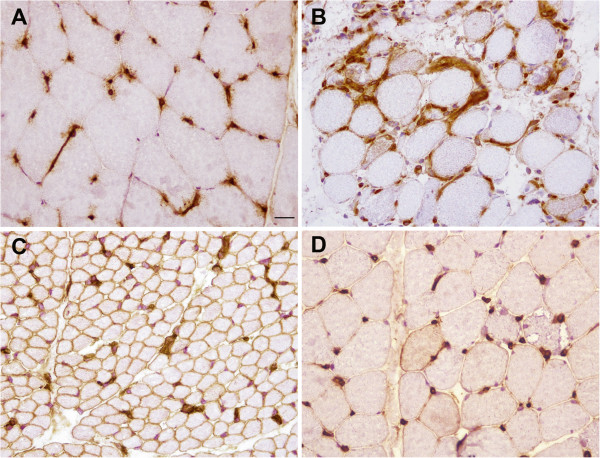
**MHC Class I antigen (HLA) expression in (A) normal muscle , (B) muscle from patient with mutations in *TK2 *(P4) (C) *SUCLA2 *(P10) and (D) in muscle bearing a large mtDNA deletion (P7).** Scale bar = 25 μm.

The inflammation gene class included components of the complement cascade such as *C3* (FC + 2.5), Major Histocompatibility Complex Class I such as *HLA-B* (FC + 2.53), major Histocompatibility Complex Class II such as *HLA-DPA1* (FC + 1.7×), interleukins (*IL17B*, FC + 2.4) and interferon-induced genes (*IFI44*, FC + 3.2 and *IFI44L*, FC + 3.7). We confirmed the induction of an immune response by immunohistochemistry using antibodies against HLA antigen (MHC-Class I) and against inflammatory cell markers in muscle biopsies from patients with *TK2* mutations (P4 and in two consecutive biopsies from an additional patient not included in the array, P11 Table 1), a patient with MDS due to *SUCLA2* mutations (P10, Table 1) and in disease controls (P5, P6 and P7 with large mtDNA deletions, Table 1). In TK2 deficient muscle HLA I was expressed in the sarcolemma and/or cytoplasm of some muscle fibres and in inflammatory cells. HLA I was detected on the sarcolemma of most fibres and in inflammatory cells in SUCLA2 deficient muscle. In muscle from patients with large mtDNA deletions, HLA I was expressed on isolated muscle fibres located near ragged red fibres (Figure 2). In all cases, the inflammatory infiltrate was mainly composed of macrophages which express CD68 (data not shown).

Within the enriched ontologies related to the regulation of cell death by apoptosis and the cell cycle we found numerous genes that were induced at comparable levels suggesting their coordinated regulation. They include genes that encode molecules that transmit apoptotic signals from the cell surface (*BID*, + FC 3.2) and others that are activated via intracellular signals such as DNA damage via the p53 pathway (osteopontin/*SPP1*, FC + 3.4; *PUMA* FC + 3.4) and genes that encode key regulators of the cell cycle such as p21 or cyclin dependent kinase inhibitor 1 (*CDKN1A*, FC + 3.7) and growth arrest and DNA-damage-inducible alpha (*GADD45A* FC + 2.2).

One of the most over-expressed genes in TK2 deficient muscle corresponds to *TRIB3* (FC + 47) which encodes a putative kinase which is induced by NFκB and which in turn inhibits the activity of the latter. TRIB3 sensitizes cells to apoptosis induced by TRAIL and TNF and it has been shown to interact with Akt1 inhibiting its pro-survival effect.

**Figure 3:**
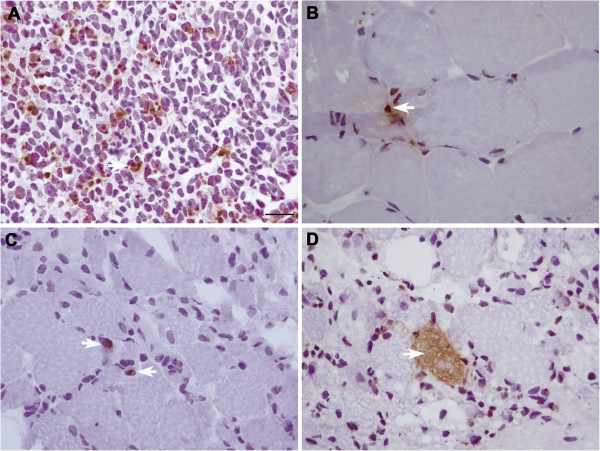
**TUNEL assay in sections from (A) Wilms tumor and muscle from (B) P7, (C,D) (P7) patients 3 and 4 with mutations in *TK2*.** Scale bar = 25 μm.

Several of the apoptotic signals above mentioned converge in the mitochondria and lead to the release of cytochrome c and the activation of Apaf-1 and Caspase-3. We investigated caspase-3 activation at the protein level by immunofluorescence in the muscle biopsies of 3 patients with *TK2* mutations (P3, P4 and P11) and other mitochondrial encephalomyopathies (P7 and P12, Table 1). As a positive control we stained frozen sections from Wilms Tumor, which is a form of paediatric nephroblastoma which shows high numbers of apoptotic cells. We observed very strong immunoreactivity against active caspase-3 in the cytoplasm of numerous fibres in TK2 deficient muscle as well as in RRF in P7 and P12 (Figure 3).

**Figure 4:**
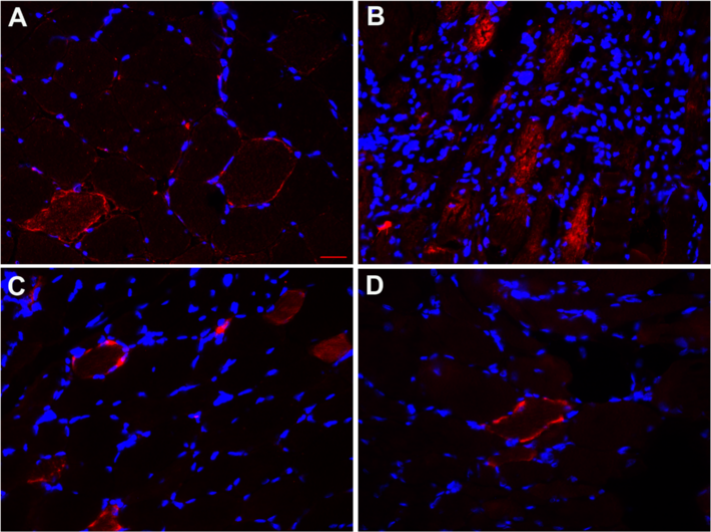
**Caspase-3 activity. (A)** P5 with a large mtDNA deletion, **(B)** P4 and **(C,D)** two consecutive biopsies of P11. Scale bar = 25 μm.

Caspase-3 activation leads to DNA fragmentation so we performed the TUNEL assay in muscle biopsies from patients with TK2 mutations (P3, P4), a patient with *SUCLA2* mutations (P10, Table 1) and patients with confirmed mitochondrial encephalomyopathy (P6, P7 and P12). We observed TUNEL positivity in myonuclei and apoptotic bodies in TK2 deficient muscle as well as in some of the myonuclei of the RRF in a patient with a MELAS phenotype (P12) (Figure 4).

Several genes encoding enzymes involved in aminoacid, urea cycle and lipid metabolism were over-expressed in response to TK2 deficiency. Noteworthy is the induction of *SHMT2* (FC + 3.4) encoding serine hydroxymethyltransferase-2 that catalyses the conversion of serine and tetrahydrofolate (THF) to glycine and methylene THF. More importantly in the context of mtDNA homeostasis, SHMT2 is one of the three key enzymes (together with thymidylate synthase (TYMS) and dihydrofolate reductase (DHFR) involved in de novo thymidylate biosynthesis pathway in mitochondria [25]. Thus, the upregulation of this pathway is likely to represent a mechanism to compensate for the lack of dTMP as a result of the severely reduced TK2 activity in these patients. *TYMS* gene expression was also increased by 1.5 fold but *DHFR* was not significantly changed.

The *ASS1* gene (FC + 4.7) encodes argininosuccinate synthetase-1, a cytosolic urea cycle enzyme expressed in most tissues. The enzyme is involved in the synthesis of arginine and catalyses that condensation of citrulline and aspartate to argininosuccinate using ATP. It is a key enzyme because it links the Krebs cycle with the urea cycle and pyrimidine synthetic pathway. Mutations in *ASS1* cause citrullinemia type I [26]. Its induction suggests an increase in the rate of ammonia detoxification and aminoacid degradation as a source of energy. Within this category we also found *ASNS* (FC + 7.9) encoding asparagine synthethase and *PSAT1* (FC + 4.4) encoding phosphoserine aminotransferase that are enzymes of the asparagine and serine biosynthetic pathways respectively.

There was an important induction of the *HMGCS2* gene (FC + 6.2) which encodes HMG-CoA synthase, as a key enzyme in ketogenesis which is a pathway to derive energy from fatty acids when blood glucose levels are low or the TCA cycle is impaired. *HMGCS2* has been shown to be expressed in skeletal muscle [27] although ketogenesis only occurs in liver cells. Thus, the significance of *HMGCS2* over-expression is unclear unless it is related to another yet unknown function. TRIB3 (PC + 47) and FGF-21 (FC + 10) are genes involved in the regulation of lipolysis upon nutrient deprivation [23].

Within the enriched GO_BPs related to glucose and glycogen metabolism we found several genes encoding for key enzymes indicating an important and coordinated attenuation of those pathways at least at the gene expression level. These included lactate dehydrogenase A (*LDHA*, FC -4) and Phosphofructokinase-fructose-biphosphatase -1 and -3 (*PFKFB1*, FC -3.8; *PFKFB3*, FC -4.6). The two latter are enzymes that regulate the levels of fructose-2,6-biphosphate which is a potent stimulator of glycolysis and an inhibitor of gluconeogenesis. Thus, the activity of these enzymes regulates glucose metabolism. PFKFB1 is ubiquitously expressed whereas PFKFB3 is mainly expressed in skeletal muscle and central nervous system. It is however worth bearing in mind that gluconeogenesis takes places mainly in liver whereas it is very little active in skeletal muscle and therefore the biochemical significance of some of the observed changes at mRNA level remain unclear. *PHKA1* (FC -3.6) and *PHKG1* (FC -3.4) encode two out of the 16 subunits of the phosphorylase b kinase enzyme which together with glycogen phosphorylase (*PYGM*, FC -4.8) regulates the degradation of glycogen to release glucose molecules.

Amongst the under-expressed components of the mitochondrial respiratory chain and related genes we found cytochrome b (*CYTB*, FC -3.3), mitochondrially encoded cytochrome c subunit II (*COX 2*, FC -3.7) and aarF domain containing kinase 3 (*ADCK3*, FC - 3.4) which is involved in the synthesis of Coenzyme Q.

Regarding genes involved in nucleotide metabolism that were under-expressed it is worth mentioning guanosine monophosphate reductase (*GMPR1*, FC -3.3) which catalyses the irreversible NADPH-dependent reductive deamination of guanosine monophosphate (GMP) to inosine monophosphate (IMP) which is the precursor of both guanine (G) and adenine (A) nucleotides. Thus, it plays an important role in maintaining the intracellular balance of purine nucleotides.

Within the calcium/ion transport gene category of under-expressed genes we found calcium channels such as plasma membrane calcium-transporting ATPase 2 (*ATP2B2*, FC -3.2), calcium channel, voltage-dependent, L type, alpha 1S subunit, (*CACNA1S,* FC -3.6), potassium channels such as ATP-sensitive inward rectifier potassium channel 12 (*KCNJ12*, FC -4) and sodium channels including Sodium channel voltage gated Type 1, beta subunit (*SCN1B,* FC -5.3) which is the channel that serves to transmit membrane action potentials in the CNS and skeletal muscle and is localised both in plasma membrane or in the mitochondrial membrane.

#### ***KEGG pathway analysis***

The DAVID functional analysis tool was used for the KEGG canonical pathways enrichment analysis using the list of differentially expressed transcripts (FDR < 0.05, Table 2). Pathways terms with a% FDR < 20 were considered as significantly enriched. We obtained results in agreement to the GO_BP analysis described above (Table 4). Amongst the over-expressed genes, 12 categories were significantly over-represented (%FDR < 20), 8 were related to immune response, 1 to aminoacid metabolism, 1 to cell adhesion, 1 to p53 signalling pathway and 1 to PPAR signalling pathway. Within the p53-signalling pathway KEGG identified three additional genes which were not identified by GO: DNA damage binding protein-2 or p48 (*DDB2*) which is expressed in a p53 dependent manner and is involved in global genome repair¸ tumor necrosis factor receptor superfamily 10B or death receptor 5 (*TNFRSF10B*) which is a TRAIL receptor which engages a caspase dependent apoptosis cascade involving the intracellular adaptor FADD and is induced by DNA damage in a p53 dependent manner and thirdly, p53 effector-related to PMP22 (*PERP*) which is a tumor suppressor gene encoding a transmembrane protein widely expressed in response to p53. For the under-expressed genes, KEGG identified 7 significantly enriched pathways that could be grouped into those related to muscle contraction (4), sugar and glycogen metabolism (3) and calcium homeostasis (1). Thus, KEGG analysis reinforced and complemented the GO data and highlighted the importance of the p53 pathway.

**Table 4:** KEGG Pathway Enrichment Analysis for differential genes in MDS vs control muscle

**KEGG Pathway**	**FDR (%)**
**Over-expressed**	
Intestinal immune network for IgA production	0.03
Systemic lupus erythematosus	0.05
Viral myocarditis	0.09
Type I diabetes mellitus	0.10
Allograft rejection	0.33
Graft-versus-host disease	0.52
Cell adhesion molecules (CAMs)	0.67
Asthma	0.92
p53 signaling pathway	2.05
Autoimmune thyroid disease	2.20
PPAR signaling pathway	2.24
Glycine, serine and threonine metabolism	8.98
**Under-expressed**	
Cardiac muscle contraction	1.16E-04
Glycolysis/gluconeogenesis	0.05
Fructose and mannose metabolism	0.09
Hypertrophic cardiomyopathy (HCM)	0.61
Dilated cardiomyopathy	1
Calcium signaling pathway	1.9
Starch and sucrose metabolism	2.4
Arrhythmogenic right ventricular cardiomyopathy (ARVC)	7.2

MDS: mitochondrial DNA depletion syndrome group. FDR: false discovery rate.

#### ***Ingenuity pathways analysis (IPA)***

We applied IPA to the list of differential genes to further explore the biological significance of the expression data. IPA predicts those affected molecular functions, the upstream regulators (transcription factors) which appear to be activated within a set/group of differential genes as well as the diseases and disorders which are likely to be associated with a given expression profile (Table 5). The top 3 molecular functions associated with TK2 deficiency consisted of cell death and survival, cellular function and maintenance (related to actin cytoskeleton) and small molecule biochemistry (removal of D-glucose). Another function predicted to be activated was immune cell trafficking (containing genes for cytokines and complement system) whereas functions related to cardiovascular and skeletal system development and function were predicted to be diminished.

**Table 5:** Biological functions associated with TK2 deficiency according to Ingenuity Pathway Analysis

**Category**	**Function**	**P-value**	**molecules**
**Diseases and disorders**			
	Cancer	1.88E-14 - 1.18E-02	253
	Neurological Disease	1.81E-11 - 8.60E-03	160
	Skeletal and muscular disorders	1.81E-11 - 8.38E-03	166
	Cardiovascular disease	3.52E-09 - 1.14E-02	102
	Hereditary disorder	6.31E-08 - 1.18E-02	98
**Molecular and cellular functions**			
	Cell death and survival	2.63E-08 - 1.11E-02	165
	Cellular function and maintenance	1.02E-05 - 1.06E-02	135
	Small molecule biochemistry	1.02E-05 - 1.18E-02	105
	Cell signaling	1.17E-05 - 1.18E-02	38
	Vitamin and mineral metabolism	1.17E-05 - 1.18E-02	31
**Physiological system development and function**			
	Skeletal and muscular system development and function	1.22E-20 - 1.18E-02	99
	Embryonic development	1.89E-05 - 1.18E-02	82
	Organ morphology	1.22E-20 - 1.18E-02	80
	Organ development	1.89E-05 - 1.18E-02	63
	Cardiovascular system Development and function	1.66E-07 - 8.20E-03	57

IPA allows the identification of the cascade of upstream transcriptional regulators (TF) that can explain the observed gene expression changes in a user’s dataset. It is based on prior knowledge of transcriptional regulators and their targets in the Ingenuity Knowledge database. When the direction of change is consistent with the literature (expression in the dataset relative to control) across the majority of targets for a given TF then the TF is predicted to be active. IPA identified TP53, TP63, HTT, TP73 and NCOA2 as the top 5 activated TF in our dataset (TK2 deficient muscle versus control muscle).

TP53, TP63 and TP73 belong to the same family and encode tumour proteins that are key regulators of cell survival and cell death. *HTT* encodes for Huntingtin which up-regulates the expression of Brain Derived Neurotrophic Factor (BDNF). The nuclear receptor coactivator 2 (*NCOA2*) is a transcriptional coregulatory protein that acylates histones, which makes downstream DNA more accessible to transcription.

**Figure 5:**
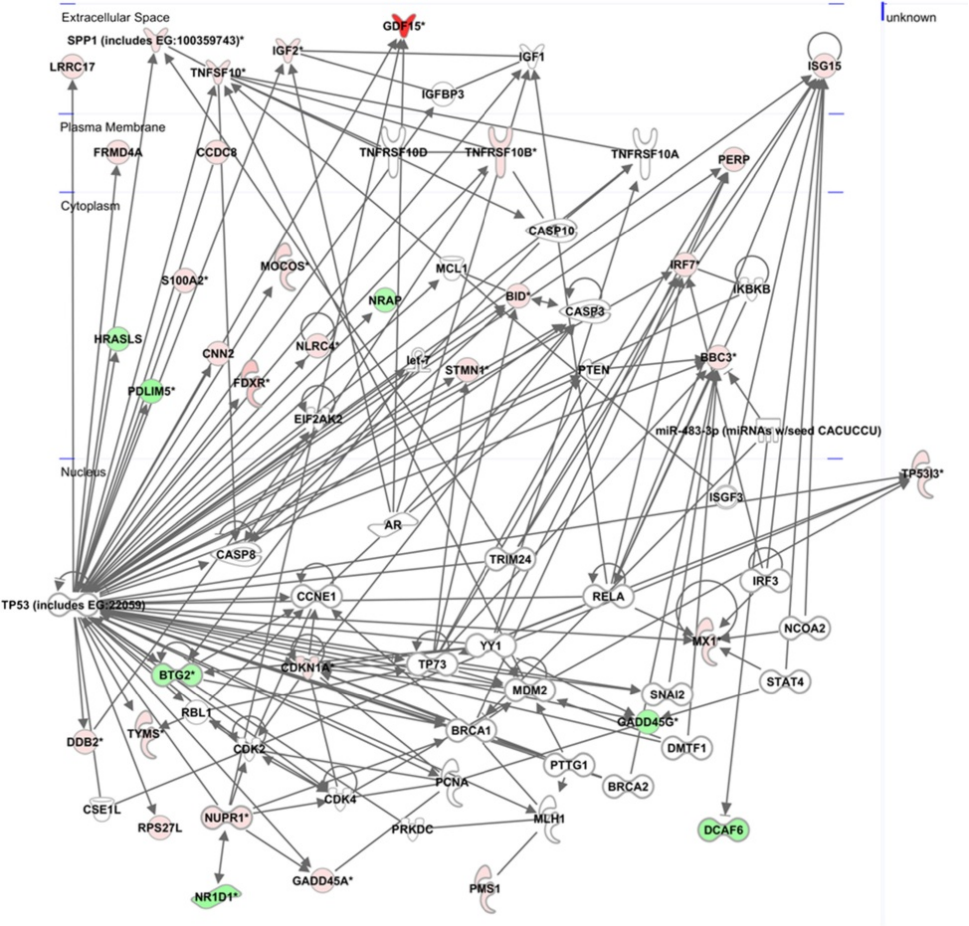
**IPA graphic representation of the network “Cell death and survival, DNA replication, recombination, and repair, cellular response to therapeutics”.** Nodes represent genes and lines show the relationship between genes. The intensity of the node colour indicates the degree of the over-expression (red) or under-expression (green) and the fold change is indicated. The cellular sub-localisation for each node, when known, is also given.

IPA identified several highly connected and well scored networks of interacting genes consisting on a number of the over-expressed and under-expressed genes identified in our study (focus genes) and others that the software predicts as also forming part of the same network. Highly interconnected networks are likely to represent significant biological function, and a score is calculated as the likelihood that focus proteins are in the network due to random chance. Of particular interest was the network “Cell Death and Survival, DNA Replication, Recombination, and Repair, Cellular Response to Therapeutics” network which is shown in Figure 5. Hub genes in this network are *PUMA/BBC3, PERP, BID, TNFSF10, TNFRSF10B, TP53I3, MX1, ISG15* as focus genes, and *TP53, RELA, MDM2, CDK2, CDK4, YY1, CASP3, CASP8* as non-focus genes. *TP53* shows the highest connectivity as clearly revealed by the representation of the network. The cellular sub-localisation for each node, when known, is also given.

### Genes differentially expressed in skeletal muscle with large mtDNA deletions versus mTDNA depleted muscle

#### ***Summary***

In order to discern those gene expression changes specific to mtDNA depletion and not due to generalised mitochondrial dysfunction we used muscle with large mtDNA deletions as a disease control (MDEL) since these can also present in children, affect several mtDNA genes and muscle samples are more widely accessible. Using the statistical test RankProd we identified 455 and 638 unique genes that were significantly under-expressed or over-expressed respectively (FDR < 0.05) in MDEL muscle relative to MDS muscle (Table 6). Thus, the expression profile of both groups of mitochondrial myopathies is markedly different. The complete list of differentially expressed genes between these two groups is shown in Additional file 3.

**Table 6:** Summary of differential genes in MDEL vs MDS muscle

**MDEL vs MDS**	**DOWN**	**UP**
FDR < 0.05	455	638
FDR < 0.05 & IPA SKM	352	501

Number of unique genes. MDEL: mitochondrial DNA deleted group. MDS: mitochondrial DNA depletion syndrome group. FDR: False Discovery Rate. IPA SKM: genes filtered by expression in skeletal muscle according to Ingenuity Pathway Analysis.

The Top 10 under-expressed and over-expressed genes in this comparison are listed in Table 7. The top 10 under-expressed genes coincide with genes which are very significantly over-expressed in TK2 deficient versus control muscle. This is the case for the markers of muscle fibre regeneration (*MYH3, MYH8* and *TNNT2*) and fibrosis (*COL19A1*). This reflects the differences in the pathology of both forms of mitochondrial disease, muscle biopsies from patients with MDEL generally do not show proliferation of connective tissue or generation and in fact CK levels are normal whereas they are elevated in MDS patients with *TK2* mutations.

**Table 7:** Top 10 under-expressed and over-expressed genes in MDEL vs MDS groups

**Gene Name**	**Description**	**FC**	**FDR**
**Under-expressed**			
MHY3	Myosin Heavy Chain Neonatal	-61.4	0
MHY8	Myosin Heavy Chain Embryonic	-32.9	0
GDF15	Growth Differentiation Factor 15	-30.4	0
SNAR-A3	Small nuclear ILF3/NF90-associated RNA A3	-15.8	0
TNNT2	Troponin T2, cardiac	-15.5	0
MYBPH	Myosin binding protein	-14.6	0
DMBT1	Deleted in malignant brain tumors 1	-11.8	0
LMF1	Lipase maturation factor 1	-10	0
COL19A1	Collagen type XIX	-9.9	0
TRIB3	Tribbles homolog 3	-9.6	0
**Over-expressed**			
FBP2	Fructose-1,6-bisphosphatase 2	18.4	0
AQP4	Aquaporin 4	17.3	0
C8ORF22		14.2	0
ATP2A1	ATPase, Ca++ transporting, cardiac muscle, fast twitch 1	10.8	0
KRTAP3-3	Keratin-associated-protein 3	10.6	0
C15ORF27		8	0
CALML6	Calmodulin like 6	8	0
DHRS7C	Dehydrogenase/reductase (SDR family) member 7C	7.9	0
MLF1	Myeloid leukemia factor 1	7.1	0
TSPAN8	Tetraspanin-8	7	0

MDEL: mitochondrial DNA deleted group. MDS: mitochondrial DNA depletion syndrome group. FC: fold-change. FDR False Discovery Rate.

Likewise, the majority of the top 10 over-expressed genes in this comparison correspond to genes which are significantly under-expressed in TK2 deficient muscle relative to controls such as *FBP*2 and *ATP2A1* highlighting the marked repression of those two genes (associated with attenuated glycolysis and loss of fast fibers) in the MDS group. There are however two genes which are significantly over-expressed in KSS relative to MDS which are not significantly altered in TK2 deficient muscle. These encode Keratin-associated-protein 3 (*KRTAP3-3*) and an EF-hand containing Ca2+ binding protein, calmodulin-like 6 (*CALML6*).

#### ***Gene ontology***

GO analysis revealed induction of several metabolic pathways (76 GO_BP categories%FDR < 20) in the MDEL group relative to MDS muscle (Additional file 4). 53 of those were the same GO_BPs that were repressed in MDS muscle relative to normal muscle and were mainly related to glucose and glycogen metabolism and skeletal muscle system. The remaining 23 GO_BPs were unique to the MDEL_MDS comparison. Out of those we were particularly interested in the 7 categories related to protein catabolism, the TCA cycle (3) and co-factor metabolism (4)

Within the protein catabolism category we found 40 unique genes. These encode several ubiquitin-specific-proteases (*UPS2, USP13, USP 15, USP24, USP25, USP38, USP47*), which are responsible for the release of ubiquitin from degraded proteins, genes encoding for ubiquitin-conjugating enzymes (*UBE2G1, UBE2D4, UBE2D1, UBE2B, UBE2E3*), which catalyse the second step in the ubiquitination process and several components of the E3 ubiquitin- ligase complex (*TRIM63, NEDD4, FEM1A, CUL3, RNF123, WWP1, DCUN1D2, RNF41*).

The TCA related GO functionalities contained 6 unique genes encoding for subunits B and C of the respiratory chain complex II (*SDHB* and *SDHC*), subunits beta of succinate-CoA ligase (*SUCLG2* and *SUCLA2*), Dihydrolipoyl dehydrogenase (*DLD*) and isocitrate dehydrogenase (*IDH2*) both of which participate in pyruvate metabolism. The co-factor metabolism category included genes encoding various enzymes and co-factors such as pantothenate kinase 4 (*PANK4*) and phosphopantothenoylcysteine decarboxylase (*PPCDC*), both of which participate in Coenzyme A biosynthesis, and COX10 which is important for assembly of complex IV of the respiratory chain.

There were 79 GO_BP categories significantly repressed in MDEL versus MDS group (Additional file 4). 56 of them were not induced in MDS group relative to control. Angiogenesis appeared to be attenuated in MDEL group relative to MDS with 17 unique genes significantly under-expressed including various collagen genes (*COL1A1, COL1A2, COL3A1, COL5A1, COL18A1*), proteoglycans (*CSPG4*), blood vessel survival factors (*ANGPTL4*), growth factors (*FGF9*) and early response genes downstream of growth factors such as *ZFP36L1*.

Thus, this GO analysis indicates that intermediate metabolism involving the TCA cycle and protein breakdown via the ubiquitin-proteasome system are significantly induced as a result of large mtDNA deletions. In contrast, angiogenesis is impaired.

#### ***KEGG pathway analysis***

KEGG identified 9 canonical pathways as significantly enriched in MDEL relative to MDS including the TCA cycle (Table 8). Only 5 canonical pathways appeared attenuated in MDEL relative to MDS amongst which we found p53 signalling and two pathways related to the immune response.

**Table 8:** KEGG Pathway Enrichment Analysis for differential genes in MDEL vs MDS muscle

**KEGG Pathway**	**% FDR**
**Over-expressed**	
hsa04260:Cardiac muscle contraction	0.09341201
hsa04020:Calcium signaling pathway	0.162733253
hsa00010:Glycolysis / Gluconeogenesis	1.309001667
hsa00020:Citrate cycle (TCA cycle)	4.872315134
hsa00260:Glycine, serine and threonine metabolism	4.872315134
hsa05012:Parkinson’s disease	6.113594087
hsa04910:Insulin signaling pathway	8.953173862
hsa05010:Alzheimer’s disease	13.57653918
hsa00190:Oxidative phosphorylation	17.58596026
**Under-expressed**	
hsa04512:ECM-receptor interaction	1.95E-04
hsa04115:p53 signaling pathway	0.221772048
hsa05322:Systemic lupus erythematosus	0.910185594
hsa04510:Focal adhesion	3.486083165
hsa04610:Complement and coagulation cascades	5.368404888

### Meta-analysis

In order to find out how many genes were differentially expressed in common in both the MDS and MDEL groups, relative to normal muscle, we applied the web based application BioVenn [28] which allows comparing and visualising gene lists. 79 unique genes (less than 10% of the 804 genes) were in common (both up and down), Table 9.

**Table 9:** List of genes differentially expressed (over and under-expressed) relative to control muscle in both MDEL and MDS muscle

**Gene Name**	**Description**	**FC MDS**	**FC MDEL**
ACTN3	Actinin, alpha 3	-6.9	-2.7
AQP4	Aquaporin 4	-6.2	+2.8
MARCO	Macrophage receptor with collagenous structure	-4	-4.1
TNMD	Tenomodulin	-4	-4.7
C8orf22	Chromosome 8 open reading frame 22	-4	+3.6
ADAMTS8	ADAM metallopeptidase with thrombospondin type 1 motif, 8	- 3.7	-5.1
AGXT2L1	Alanine-glyoxylate aminotransferase 2-like 1	-3.5	-2.5
SLITRK4	SLIT and NTRK-like family, member 4	-3.3	-3.4
FNDC1	Fibronectin type III domain containing 1	-3.2	-2.5
SERPINA5	Serpin peptidase inhibitor, clade A (alpha-1 antiproteinase, antitrypsin), member 5	-2.8	-2.4
GAS2L2	Growth arrest-specific 2 like 2	-2.8	-2.6
MYH1	Myosin, heavy chain 1, skeletal muscle, adult	-2.7	+2.8
IL32	Interleukin 32	-2.6	-4
ITGA10	Integrin, alpha 10	-2.5	-3.3
FMOD	Fibromodulin	-2.3	-4
FOS	FBJ murine osteosarcoma viral oncogene homolog	-2.3	+2.4
IL17D	Interleukin 17D	-2.3	+2.6
EIF1AY	Eukaryotic translation initiation factor 1A, Y-linked	-2.2	-2.5
RPS4Y1	Ribosomal protein S4, Y-linked 1	-2.1	-2.7
GDF15	Growth and differentiation factor 15	+187.9	+6.2
MYH8	Myosin heavy chain embryonic	+118.7	+3.6
TRIB3	Tribbles homologue 3	+47.1	+5
DEFB1	Defensin beta	+16.6	+4.5
Tmem63c	Transmembrane protein 63C	+16.2	+2.9
SNORD3B-1	Small nucleolar RNA, C/D box 3B*-1*	+6.8	+3.2
SNORD15A	Small nucleolar RNA, C/D box 15A	+6.3	+3.7
SNORA28	Small nucleolar RNA, H/ACA box 28	+6.3	+4.9
ATF5	Activating transcription factor 5	+6.3	+2.1
HMGCS2	3-hydroxy-3-methylglutaryl-CoA synthase 2 (mitochondrial)	+6.2	+2.7
GOS2	G0/G1 switch regulatory protein	+6.1	+4.8
HBA2	Hemoglobin, alpha 2	+5.9	+4.5
XIST	X (inactive)-specific transcript (non-protein coding)	+5.8	+4.1
HBD	Hemoglobin, delta	+5.8	+5.3
C3	Complement component 3	+5.6	+2.1
SNORA74A	Small nucleolar RNA, H/ACA box 74A	+5.6	+2.8
HBB	Hemoglobin, beta	+5.5	+4.9
SNORD17	Small nucleolar RNA, H/ACA box 17	+5.2	+2.5
PRODH	Proline dehydrogenase (oxidase) 1	+5.2	+2.3
CD36	*CD36* molecule (thrombospondin receptor)	+4.7	+2.4
CES1	Carboxylesterase 1	+4.6	+2.9
SCL26A9	Solute-linked carrier 26 alpha 9	+4.4	+3.6
FABP5	Fatty acid binding protein 5	+4.4	+2.3
RNU105A	RNA, U105A small nucleolar	+4.3	+2.2
RN7SK	RNA, 7SK small nuclear	+4.3	+2.7
CIDEC	Cell death-inducing DFFA-like effector c	+3.9	+2.6
FABP4	Fatty acid binding protein 4	+3.9	+2.1
IGLL5	Immunoglobulin lambda-like polypeptide 5	+3.9	+3
ALAS2	Aminolevulinate, delta-, synthase 2	+3.3	+2.6
MYL10	Myosin, light chain 10, regulatory	+3.2	+4
ADH1A	Alcohol dehydrogenase 1A (class I), alpha polypeptide	+3.1	+3.5
SCARNA5	Small Cajal body-specific RNA 5	+2.4	+2.4
SNORA81	Small nucleolar RNA, H/ACA box 81	+2.3	+2.3
KCNE1L	KCNE1-like	+3	+2.6
RBP4	Retinol binding protein 4	+2.9	+3
GZMH	Granzyme H (cathepsin G-like 2, protein h-CCPX)	+2.9	+2.3
HBG1	Hemoglobin, gamma A	+2.7	+3.6
DPYSL4	Dihydropyrimidinase-like 4	+2.6	+3.1
FMO2	Flavin containing monooxygenase 2	+2.5	+2.6
C20orf26	Chromosome 20 open reading frame 26	+2.5	+3.5
HIST1H4L	Histone cluster 1, H4l	+2.5	+2.7
SCARNA16	Small Cajal body-specific RNA 16	+2.5	+3.9
COL21A1	Collagen, type XXI, alpha 1	+2.4	+2.7
ANGPTL4	Angiopoietin-like 4	+2.4	-3.5
RNU1-5	RNA, U1 small nuclear *5*	+2.3	+3.8
COL1A1	Collagen, type I, alpha 1	+2.1	-4.2
RNU2-2	RNA, U2 small nuclear *2*	+2	1.9

These included GDF-15, haemoglobin genes (*HBA2, HBB* and *HBD*), inflammation related genes (*C3, IL17D, IL32, DEFB1*), collagens (*COL1A1, COL21A1*), fatty acid transport (*CD36, FABP4* and *FAB5*). In most cases the direction of the change was the same (up or down) but to a different degree. For example, in MDS muscle *ACTN3* was under-expressed by almost 7 fold whereas in MDEL it was under-expressed by 3 fold. However, for a few genes the direction of the change was opposite. This is the case for aquaporin 4 (*AQP4*) which was under-expressed (FC -6.2) in MDS muscle whereas it was over-expressed in MDEL muscle (FC + 2.8) and also for *ANGPTL4, COL1A1, C8orf22, MYH1* and *IL71D* (Table 8).

### Validation of changes in expression

We selected some of the most significant and markedly differential genes to measure their expression levels using a quantitative PCR approach. The TK2 deficient group included three of the four samples used in the array (P2, P3 and P4). The disease control MDEL group included three of the four samples with large mitochondrial DNA deletions used in the array (P5, P6 and P7) and an additional sample (P9, Table 1). In addition we studied one patient with mutations in *SUCLA-2* (P10, Table 1). Results were expressed relative to TATA box binding protein (*TBP*) and hypoxanthine phosphoribosyltransferase 1 (*HPRT1*) as endogenous control genes to normalize transcription levels amongst patients. Results were analysed with qBase^plus^ software. FC above or below 1.5 were considered significant.

Differential expression was confirmed for all the selected genes (Table 10) although the dynamic range of differential gene expression was different for the two techniques. This was particularly true for the genes with the highest levels of over-expression, *GDF-15, TRIB-3, COL19A1* and *FGF21*. Most genes that were changed in TK2 deficient muscle were also changed in the same direction in muscle with SUCLA2 defects, for example, the glycolytic genes *FBP2, PFKFB3* and *LDHA*. However, there were some differences such as cytochrome b which was over-expressed instead of under-expressed as in TK2 deficient muscle.

**Table 10:** Validation of microarray results with real time PCR

		**MDS**		**MDEL**		**SUCLA-2**
**Gene**	**Description**	**FC array**	**FC Fluidigm**	**FC array**	**FC Fluidigm**	**FC Fluidigm**
FBP2	fructose-1,6-bisphosphatase 2	-9.1	-23.4	ns	-1.18	-1.59
ATP2A1	ATPase, Ca++ transporting, cardiac muscle, fast twitch 1	-9.1	-13.3	ns	1.07	1.31
PFKFB3	Phosphofructokinase-biphosphatase-3	-4.6	-1.7	ns	-1.14	-2.32
LDHA	Lactate-dehrydrogenase-A	-4.1	-7.9	ns	-1.67	-2.85
ADCK3	aarF-domain-containing kinase 3	-3.4	-2.7	ns	-1.21	1.19
CYTB	Cytochrome b	-3	-7.63	ns	-1.32	3.44
GDF15	Growth Differentiation Factor 15	188	845	6	154.6	2.46
TRIB3	Tribbles homolog 3	47	141.3	5	29.5	2.92
TNNT2	Troponin T2, cardiac	21	35.9	ns	7.3	6.62
COL19A1	Collagen type XIX	11	100.6	ns	19.7	1.66
FGF21	Fibroblast Growth Factor 21	10.8	1281.1	ns	286.8	-
HMGCS2	3-hydroxy-3-methylglutaryl-CoA synthase 2	6.2	9.2	2.7	4.99	66.85
PTPRF	Receptor-type tyrosine-protein phosphatase F	6.1	9.6	ns	2.09	3.2
G0S2	G0/G1 switch regulatory protein	6.1	1.91	4.8	2.21	1.5
SHMT2	Serine hydroxyl-methyltransferase 2	3.4	4.8	ns	1.84	-1.06
ASS1	argininosuccinate synthase-1	4.7	3.1	ns	1.53	3.52
TP53I3	TP53 induced gene 3	3.5	2.42	ns	-1.30	1.54
DPYSL4	Dihydropyrimidinase-related protein 4	2.6	5.48	3.1	1.30	-
TP53		ns	1.8	ns	1.01	1.61
PPARG	Peroxisome proliferator-activated receptor gamma	ns	-1.02	ns	-1.10	1.54
SUCLA2	succinate-CoA ligase, ADP-forming, beta subunit	ns	-2.28	ns	-1.29	-1.35
TK2	Tymidine kinase 2	ns	-1.19	ns	-1.24	1.37

To confirm that muscle cells are able to transcribe some of the mRNAs of interest we performed qRT-PCR using RNA from the immortalised skeletal muscle cell line LHCN-M2 (kind gift of W. Wright, UT Southwestern Medical Centre, Dallas, US, [29] after differentiation for 5 days as well as from dermal fibroblast cultures. We found that both muscle cells and fibroblast express similar levels of *GDF-15* transcript (based on mean CT values) whereas fibroblast express higher levels of *TRIB3* than muscle cells. When compared to normal muscle tissue, muscle cultures expressed significantly higher levels of *GDF-15* but comparable levels of *TRIB3* (data not shown). Thus, the source of GDF-15 could be the muscle cells themselves as opposed to the fibroblasts or other cell types present in the tissue.

### Expression of GDF-15 as a possible biomarker of mitochondrial disease

**Figure 6:**
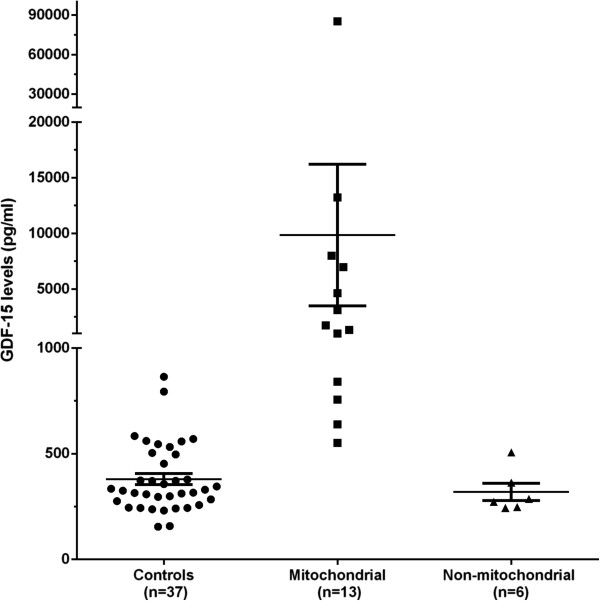
GDF-15 serum levels as measured by ELISA (pg/ml) in unaffected children (circles) and children with mitochondrial myopathies (squares).

Growth and differentiation factor-15 (GDF-15), also known as macrophage inhibitory cytokine-1 (MIC-1), is a member of the transforming growth factor beta (TGF-β) superfamily. To evaluate the potential application of GDF15 as a biomarker for mitochondrial diseases we conducted a small pilot study and measured its serum levels by ELISA. In healthy children (n = 37) the mean concentration of GDF-15 was 380.5 pg/mL and the normal range (mean ± 2 SD) between 59 and 701 pg/mL which is comparable to what has been described in other studies [30,31]. We studied 13 patients with molecularly confirmed mitochondrial myopathy and although the levels of GDF-15 were highly variable they were on average very significantly increased relative to the control group (mean 3562 [SD 3973] pg/mL) (p = 1.19673E-05) (Figure 6). The highest levels were detected in two patients with *POLG* mutations (85252 pg/mL and 13215 pg/mL) followed by P4 of this study carrying *TK2* mutations (8000 pg/mL) and a patient with lactic acidosis and epilepsy bearing the mtDNA A3243G mutation (P12), (6999 pg/mL). GDF-15 serum levels were within the normal range (mean 320 pg/mL) in 6 children with muscular dystrophy (DMD n = 3 and Ullrich Congenital Muscular Dystrophy n = 3).

In addition we measured GDF-15 levels in the conditioned medium from cultures of LHCN-M2 myotubes and normal dermal fibroblasts. We were able to detect GDF-15 in both muscle and fibroblast cultures indicating that both cell types are able to secrete it. Levels in muscle cultures were higher than in fibroblasts (804 pg/mL and 150 pg/mL respectively).

## Discussion

High-sensitivity microarray technology provides a useful tool to study changes in the whole transcriptome of skeletal muscle samples in relation to specific genetic or biochemical mitochondrial defects [32]. This is the first report of global gene expression analysis of TK2 deficient human skeletal muscle. There is a previous study of gene expression in fibroblasts from patients with TK2 or dGUOK mutations [33]. Using this approach we have detected significant changes in a set of over 700 genes which represents the adaptative response of skeletal muscle cells to TK2 defects and the subsequent severe mtDNA depletion. Furthermore, we have also identified changes in common with another form of mitochondrial myopathy that also affects various mtDNA encoded components of the respiratory chain. These two pathologies only share a small proportion of differential genes compared with normal muscle which indicates that the cell responds differently to various forms of mitochondrial dysfunction and that the changes that we have observed are specific to TK2 deficiency.

Our data indicate that TK2 deficiency results in important changes in genes involved mainly in metabolism, regulation of the cell cycle and mitochondrial DNA maintenance, oxidative stress and apoptosis.

### Metabolism

We found over-expression of several transcripts involved in the starvation response. These include *ASNS, PSAT1, FGF-21* and *TRIB3*. These genes, which were also over-expressed in twinkle deficient mouse skeletal muscle [34], have in common the presence of an amino acid response element (AAREs) in their promoter region where transcription factors of the ATF family bind to induce their expression upon limited nutrition [35]. Thus, these results suggest that TK2 deficiency induces a starvation-like response which alters lipid and aminoacid metabolism.

Two out of the three key enzymes of the de novo thymidine nucleotide synthesis pathway were co-ordinately over-expressed in the array, SHMT2 and TYMS. Coordinated expression of both proteins, but not of the third enzyme in the pathway, dihydrofolate reductase (DHFR), has been previously reported [36]. This may represent a compensatory mechanism for defective salvage pathway due to the severe reduction of TK2 activity. Alternatively, it may reflect the fact that there is an increase in regenerating cycling muscle cells which rely on de novo synthesis as opposed to non-regenerating muscle which is considered post-mitotic and thus depends mainly on the salvage pathway.

### P53, mitochondrial DNA maintenance, cell cycle and oxidative stress

*TP53* gene encodes p53, a tumor suppressor gene which is activated by several forms of cellular stress such as DNA damage, oxidative stress, ribonucleotide depletion and mitochondrial dysfunction as well as by other stimuli [37]. P53 plays a crucial role in the regulation of the cell cycle, DNA repair and apoptosis [38]. Under conditions of oxidative stress, p53 traslocates to mitochondria where it binds the mitochondrial DNA polymerase (Pol γ) and the mitochondrial transcription factor A (TFAM gene) and promotes base excision repair and/or replication of mtDNA. Treatment of cells with N-acetylcysteine (anti-oxidant) prevents traslocation of p53 to mitochondria [39].

In the present study we have demonstrated activation of several genes and pathways regulated by p53. *PIG3*, encoding TP53I3, a NADPH dependent medium chain dehydrogenase reductase (quinone reductase) was amongst the over-expressed p53 targets. *PIG3* expression can also be activated by p63 and p73 (which are also predicted to be activated in our data set) [40]. TP53I3 plays a dual role. On the one hand as a ROS producer upon induction by p53 to promote apoptosis and on the other hand as a DNA breaks sensor that activates the DNA damage response pathway (DDR pathway) after translocation to the nucleus. In normal cells subjected to genotoxic stress, the DDR pathway stabilises p53 which induces PIG3 transcription. If the damage is not excessive PIG3 produces certain amounts of ROS insufficient to induce the expression of other pro-apoptotic genes but also promotes DNA repair. If the damage cannot be repaired very high levels of PIG3 produce very high levels of ROS and finally trigger apoptosis [40]. It is interesting to mention that TP53I3 gene is within a genomic region identified by Calvo et. al as being linked to mitochondrial disease [4]. Although mutations in *TP53I3* gene have not been identified in humans, a role for cancer predisposition of certain polymorphisms has been proposed.

We found over-expression of cyclin dependent kinase inhibitor 1 (*CDKN1A*) and growth arrest and DNA-damage-inducible alpha (*GADD45A*) both of which are known to prevent progression of the cell cycle at the G1 and G2 level respectively and are downstream targets of p53 [41] (Zhang and Chen 2008).

Thus, we hypothesise that mtDNA depletion leads to p53 activation to promote mtDNA replication and repair both via its role as a transcription factor and via its interaction with Pol γ and mtTFA in mitochondria.

On the other hand, mitochondrial dysfunction and subsequent reactive oxygen species accumulation amplifies the p53 signalling cascade which induces the expression of pro-apoptotic factors, activation of caspase-3 and apoptosis in damaged cells.

The p53 pathway has been previously associated with mitochondrial DNA depletion. On the one hand, p53 deficient mouse and human fibroblasts show reduced mtDNA copy number [42].On the other hand, mutations in *RRM2B* cause a severe subtype of MDS which mainly affects skeletal muscle and kidney [7]. *RRM2B* encodes the R2 subunit of the p53-inducible ribonucleotide reductase which converts ribonucleoside diphosphates into the corresponding deoxyribonucleosides which is essential for DNA repair [43]. Mutations in *RRM2B* null mice suffer from renal failure, growth retardation, and early mortality and show decreased mtDNA content in kidney, muscle and liver [43]. In the present study we did not find increased expression of *RRM2B* but its activation via post-transcriptional mechanisms cannot be excluded.

Progressive muscle fibre loss is a feature of muscle biopsies from patients with TK2 defects. Apoptosis has been described in various neuromuscular conditions [44] including some mitochondrial myopathies [45]. Mitochondria play a key role regulating the susceptibility of cells to undergo apoptosis because they are involved in oxidative stress, Ca^2+^ homeostasis and energy supply and several of the pro- or anti-apoptotic factors localise to mitochondria. In the present study we have demonstrated for the first time evidence of over-expression of several pro-apoptotic factors (some of which are regulated by p53), caspase-3 enzymatic activation and DNA fragmentation in human skeletal muscle deficient for TK2. Anti-apoptotic drugs have been shown to prevent apoptosis, ameliorate muscle histology, slow loss of muscle fibres and increase body weight and survival in animal models for congenital muscular dystrophy and some of these drugs are under consideration for clinical trials in humans [46]. In view of our results, the use of such compounds may also be beneficial in TK2 deficient muscle to prevent rapid muscle loss.

### P53, glucose metabolism and oxidative phosphorylation

In addition to its role as “guardian” of the genome, p53 is emerging as a key regulator of many metabolic processes including glycolysis, oxidative phosphorylation, insulin sensitivity and mitochondrial stability [47]. P53 induces the expression of TP53-induced glycolysis and apoptosis regulator (TIGAR) which affects FBP2 (fructose-2,6-biphosphatase), which was under-expressed at the RNA level in our microarrays, and limits the activity of phosphofructokinase 1 thus lowering the rate of glycolysis. In this way, some glycolytic intermediates can be utilized in the pentose phosphate pathway which feeds into de novo nucleotide synthesis. P53 is also known to induce the expression of Apoptosis Inducing Factor (AIF), which is essential for complex I function [47] and of its mitochondrial homologue *AIFM2* which was over-expressed (FC + 2.6) in our microarrays Thus, it would seem that in the context of TK2 deficiency, p53 may be inhibiting glycolysis and promote de novo nucleotide synthesis. We cannot exclude that some of the observed changes are associated with selective loss of type II fibres.

### GDF-15

In humans, GDF-15 is predominantly expressed in the placenta, with low levels in the kidney, pancreas, and prostate. However, its expression can be rapidly induced by cytokines such as interleukin-1 and TGF-β [48]. GDF-15 has diverse biological functions. Early studies have shown that low serum GDF-15 levels correlate with miscarriages, indicating that it might be able to suppress inflammation in early pregnancy [49]. GDF-15 also plays an important role in tumorigenesis and metastasis since it is dramatically increased in many types of cancers [50]. The expression of GDF-15 is strongly induced by p53 and anti-tumorigenic agents, such as the non-steroidal anti-inflammatory drugs [51]. Serum GDF-15 concentrations have been shown to be also associated with the risk of acute coronary syndrome as well as its prognosis. GDF-15 can be produced by cardiac myocytes in response to ischemia, nitrosative or oxidative stress and angiotensin II [22].

*GDF-15* mRNA was dramatically increased (by almost 200×) in TK2 deficient muscle and it was also elevated in mtDNA deleted muscle (by 6 fold). We confirmed its over-expression by qRT-PCR in muscle tissue and also in the LHCM-N2 cell line demonstrating that skeletal muscle cells can transcribe this gene. Using ELISA we demonstrated that LHCN-M2 synthesise and secrete GDF-15 to the extracellular space. Thus, GDF-15 could be produced by muscle cells in vivo in response to oxidative stress and p53 activation. However, contribution from other cell types cannot be excluded.

We conducted a small pilot study to evaluate the potential application of GDF-15 as a biomarker of mitochondrial disease by measuring its serum concentration by ELISA. We established the normal range in our population of healthy children and studied serum from patients with genetically confirmed mitochondrial disease. On average, GDF-15 levels in patients were 9 times higher than in the healthy control group. Two patients had values that fell within the broad normal range that we established (mean ± 2SD). Nonetheless the GDF-15 concentration was 1.7 and 1.5 times higher than the normal mean value and would fall within a smaller range (mean ± SD). We also included 6 non-mitochondrial myopathies children with severe dystrophies whose GDF-15 values were normal. We are currently extending this study to include more patients with mitochondrial diseases (with and without myopathy), non-mitochondrial myoapthies and various neurometabolic disorders, to correlate GDF-15 plasma levels with other biochemical markers (in particular FGF-21, lactate, pyruvate, lactate to pyruvate ratio and creatine kinase values) [52] as well as with muscle histopathological markers such as COX negative and/or ragged-red fibres.

In spite of the limited number of patients and disease controls and the necessity for a larger study, the mRNA and protein data strongly suggest that GDF-15 (alone or in combination with other markers) may serve as a biochemical marker to aid in the diagnosis of mitochondrial diseases.

## Conclusions

The integrated use of Gene Ontology, KEGG pathways, IPA databases and statistical and visualization tools has allowed us to place the differential gene expression data obtained from the microarrays into a coherent functional context. Our data suggest that mtDNA depletion due to TK2 defects leads to activation of p53 signalling pathway. P53 induction may represent a compensatory mechanism to promote DNA repair and replication in mitochondria. In addition, p53 inhibits glycolysis to promote the pentose synthetic pathway. The inability of some muscle cells to replenish the dNTPs pools and overcome the loss of mtDNA beyond a certain level (despite the observed upregulation of two key enzymes of the de novo synthesis pathway) is likely to result in sustained ROS production by TP53I3 and activation of other pro-oxidative and pro-apoptotic factors which lead to the death of a proportion of muscle fibres. P53 and oxidative stress probably induce the expression and secretion of GDF-15 into the circulation. This finding may be exploited as a tool for diagnosis of mtDNA depletion and other mitochondrial myopathies although further studies are required.

## Methods

### Ethics statement

This work has been approved by the Ethical Committee of “Fundació Sant Joan de Déu”.

Written informed consent for research was obtained from all patients (or their parents/guardians) according to the Hospital Sant Joan de Déu forms and regulations.

### Muscle biopsies

Open muscle biopsies (from left quadriceps or deltoid muscles) were performed, oriented and frozen according to standard procedures [53]. Lower limb muscle samples were obtained from children non-affected by a neuromuscular disease (control muscle group) who underwent orthopaedic surgery at the Hospital Sant Joan de Déu.

### RNA preparation

Total RNA was extracted from biopsies (30 mg) or cells with RNeasy Fibrous Tissue mini kit (Qiagen, Hilden, Germany). Muscle tissue was homogenized with TissueRuptor (Qiagen, Hilden, Germany) following the manufacturer’s instructions. Quantity and quality of RNA obtained was determined with Nanodrop 8000 Spectrophotometer (Thermo Scientific, Schwerte, Germany) and its integrity with Agilent 2100 Bioanalyzer (Agilent technologies, Waldbronn, Germany). In all samples RNA integrity numbers (RIN) were >7 and 260/280 ratios near 2.0. 0.15 mg of RNA was retro-transcribed with SuperScript III First-Strand Synthesis Super-Mix for qRT-PCR (Invitrogen™, Carlsbad, CA, USA) to obtain cDNA and then was amplified with Taqman PreAmp Master Mix (Applied Biosystems, Foster City, CA, USA) following manufacturer’s instructions.

### Microarray experiments

Cyanine-3 (Cy3) labeled cRNA was prepared from 100 ng of RNA using the LowInputQuick Amp Labeling kit (Agilent technologies, Waldbronn, Germany) according to the manufacturer’s instructions, followed by RNAeasy column purification (Qiagen, Hilden, Germany). Dye incorporation and cRNA yield were checked with the NanoDrop ND-1000 Spectrophotometer. 600 ng of Cy3-labelled cRNA (specific activity >10.0 pmol Cy3/ug cRNA) was fragmented at 60°C for 30 minutes in a reaction volume of 25 ul containing 1× Agilent fragmentation buffer and 2× Agilent blocking agent following the manufacturer’s instructions. On completion of the fragmentation reaction, 25ul of 2× Agilent hybridization buffer was added to the fragmentation mixture and hybridized to Agilent SurePrint G3 Human Gene Expression 8 × 60K arrays (G4851A-028004, Agilent technologies, Waldbronn, Germany) for 17 hours at 65°C in a rotating Agilent hybridization oven. After hybridization, microarrays were washed 1 minute at room temperature with GE Wash Buffer 1 and 1 minute with 37°C GE Wash buffer 2 (Agilent technologies, Waldbronn, Germany). Scanned on an Agilent G2539A scanner at 3um resolution and 100%PMT. The intensity data of each individual hybridization were extracted and the quality was assessed with the Feature Extraction software 10.7 (Agilent technologies, Waldbronn, Germany). Raw data was corrected for background noise using the *Normexp* method. *Quantile* normalization method was applied to assure comparability across samples [54].

### Microarray statistical analysis

Statistical differential gene expression analysis between groups was performed by the non-parametric approach *Rank Prod*[55] which detects genes that are consistently highly ranked in a number of replicate experiments, a method that has shown robustness to outliers and being particularly powerful when small number of replicates is available. Those oligonucleotides that present changes between groups with FDR (false discovery rate) value lower than 0.05 were considered significant. The tool DAVID [24] was used for the calculation of the functional over-representation statistics of the different lists of significant genes obtained with *Rank Prod* analysis. Gene Ontology Biological Process and KEGG pathways data bases (Kyoto Encyclopedia of Genes and Genomes, Kanehisa, Goto et al., 2002) were considered. Also, interaction networks have been constructed using the Ingenuity Pathways Analysis tool (IPA, http://www.ingenuity.com), based on extensive records maintained in the Ingenuity Pathways Knowledge Base (IPKB).

### Real time quantitative RT-PCR

High-throughput real-time qPCR was performed according to the manufacturer’s protocol on the BioMark 48.48 Dynamic Array (Fluidigm^^®^^, South San Francisco, CA, USA) with Taqman Gene Expression Assays (Applied Biosystems, Foster City, CA, USA), (Table 11). qRT-PCR was run in triplicates in all samples and *TBP* and *HPRT1* were used as endogenous control genes to normalize transcription levels amongst patients. Results were analyzed with qBase^plus^ software (Biogazelle, Zwijnaarde, Belgium).

**Table 11:** qRT-PCR primers

**Gene name**	**Gene symbol**	**Taqman gene expression assays**
TATA box binding protein	TBP	Hs99999910_m1
Hypoxanthine phosphoribosyltransferase 1	HPRT1	Hs02800695_m1
Actin, beta	ACTB	Hs01060665_g1
Desmin	DES	Hs00157258_m1
3-hydroxy-3-methylglutaryl-CoA synthase 2 (mitochondrial)	HMGCS2	Hs00985427_m1
Serine hydroxymethyltransferase 2 (mitochondrial)	SHMT2	Hs00193658_m1
Argininosuccinate synthase 1	ASS1	Hs01597989_g1
Tumor protein p53 inducible protein 3	TP53I3	Hs00153280_m1
Complement component 3	C3	Hs00163811_m1
Tumor protein p53	TP53	Hs01034249_m1
Hemoglobin, beta	HBB	Hs00758889_s1
G0/G1switch 2	G0S2	Hs00274783_s1
Collagen, type XIX, alpha1	COL19A1	Hs00156940_m1
Protein tyrosine phosphatase, receptor type, F	PTPRF	Hs00892965_m1
Peroxisome proliferator-activated receptor gamma	PPARG	Hs01115513_m1
Fructose-1,6-bisphosphatase 2	FBP2	Hs00427791_m1
Cytochrome b	MT-CYB	Hs02596867_s1
Lactate dehydrogenase A	LDHA	Hs00855332_g1
aarF domain containing kinase 3	ADCK3	Hs00220382_m1
Troponin T type 2 (cardiac)	TNNT2	Hs00165960_m1
ATPase, Ca++ transporting, cardiac muscle, fast twitch 1	ATP2A1	Hs01092295_m1
Dihydropyrimidinase-like 4	DPYSL4	Hs01067761_m1
Fibroblast growth factor 21	FGF21	Hs00173927_m1
Tribbles homolog 3 (Drosophila)	TRIB3	Hs01082394_m1
Growth differentiation factor 15	GDF15	Hs00171132_m1
6-phosphofructo-2-kinase/fructose-2,6-biphosphatase 3	PFKFB3	Hs00998700_m1
Succinate-CoA ligase, ADP-forming, beta subunit	SUCLA2	Hs00605838_g1
Thymidine kinase 2, mitochondrial	TK2	Hs00177950_m1

### Immunohistochemistry and immunofluorescence

Cryosections (7 μm thick) were labelled without prior fixation using primary antibodies diluted in PBS 0.1% Tween-20. Immunofluorescence was visualised with species-specific secondary antibodies directly linked to Alexa-fluorophores (Molecular Probes, Eugene, OR, USA). Nuclei were visualised using DAPI (Molecular Probes, Eugene, OR, USA) diluted in PBS 0.1% Tween-20. Immunohistochemistry was performed using Novo-Link peroxidase detection kit according to manufacturer’s instructions (Novo-Link peroxidase detection kit, Leica Microsystems, Wetzlar, Germany). We used mouse monoclonal antibody against MHC Class I (Dako, Glostrup Denmark, Cat. M0736, 1:1000) and rabbit polyclonal antibody against active caspase-3 (Pharmingen, San Diego, CA, USA, Cat. 559565, 1:400). Non-specific labelling was assessed using sections incubated without primary antibodies. Immunofluorescence was visualised under a conventional epifluorescence microscope (Leica DM5000B). Images were acquired with Leica Imaging Suite (Leica Microsystems, Wetzlar, Germany) and were quantified with ImageJ software (http://rsbweb.nih.gov/ij/).

### TUNEL Assay

The TUNEL assay was employed to examine apoptosis via DNA fragmentation using the ApopTag Plus Peroxidase In Situ Apoptosis Detection Kit (Millipore Corporation, Billerica, MA, USA, Cat. S7101) according to manufacturer’s instructions.

### GDF-15 serum levels

We analysed serum samples collected from patients and controls of paediatric age. We measured GDF-15 concentration in duplicate samples using the human GDF-15 Quantikine ELISA kit (R&D Biosystems, Minneapolis, US) according to Manufacturer’s instructions. The values from each assay were extrapolated from a standard four parameter logistic (4-PL) curve fit. Since serum samples were diluted, the concentration read from the standard curve was multiplied by 4 to obtain the final pg/mL concentration.

### Statistical analysis

Average values are expressed as mean ± standard error (MEAN ± SE). Significance was tested by Student unpaired *t*-test and *P*-value <0.05 was considered as significant (NS, non-significant, *P < 0.05, **P < 0.01 and ***P < 0.001).

### Availability of supporting data

Gene expression data has been deposited at the National Centre for Biotechnology Information Gene Expression Omnibus (GEO) database as GEO Series accession number GSE43698.

## Abbreviations

mtDNA: Mitochondrial DNA; MDS: Mitochondrial DNA depletion syndrome; TK2: Thymidine kinase; FDR: False-discovery-rate; FC: Fold-change; GO: Gene ontology; IPA: Ingenuity Pathway Analysis; GDF-15: Growth and differentiation factor 15; CPEO: Chronic progressive external opthalmoplegia; KSS: Kearns-Sayre syndrome.

## Competing interest

The authors declare that they have no competing interests.

## Authors’ contributions

Contributions to study conception and design, data acquisition, analysis and interpretation, draft preparation, revision and approval: KSG, PS, JMC. Contributions to data acquisition, analysis and interpretation, draft preparation, revision and approval: JC, FI, AR, BDP, NA, OC, CJ Contributions to data acquisition, analysis and interpretation, draft revision and approval: MM, RM, JVM, SM, MM, FG, DML, RQM, MF, MJ, LGE, RPE, MR, TF. All authors read and approved the final manuscript.

## Supplementary Material

Additional file 1 Complete list of differential genes (FDR < 0.05) in MDS muscle versus control muscle. Click here for file

Additional file 2 GO_BP terms significantly enriched (%FDR < 20) in MDS muscle versus control muscle. Click here for file

Additional file 3 Complete list of differential genes (FDR < 0.05) in MDEL muscle versus MDS muscle. Click here for file

Additional file 4 GO_BP terms significantly enriched (%FDR < 20) in MDEL muscle MDS muscle. Click here for file
